# The Study of Soil Bacterial Diversity and the Influence of Soil Physicochemical Factors in Meltwater Region of Ny-Ålesund, Arctic

**DOI:** 10.3390/microorganisms10101913

**Published:** 2022-09-27

**Authors:** Long Wang, Jie Liu, Jialin Yuan, Nengfei Wang

**Affiliations:** 1 Department of Bioengineering, College of Marine Sciences and Biological Engineering, Qingdao University of Science and Technology, Qingdao 266042, China; 2 School of Chemistry and Chemical Engineering, Linyi University, Linyi 276012, China; 3 First Institute of Oceanography, Ministry of Natural Resources, Qingdao 266061, China

**Keywords:** Arctic Ny-Ålesund, meltwater area, soil bacterial community, soil physicochemical properties, high-throughput sequencing

## Abstract

Global climate change has caused the changes of the ecological environment in the Arctic region, including sea ice melting, runoff increase, glacial lake expansion, and a typical meltwater area has formed in the Arctic coastal area. In this study, the meltwater areas near six different characteristic areas of Ny-Ålesund in 2018 were taken as the research objects, and high-throughput sequencing of V3–V4 regions of all samples were performed using 16S rDNA. Among the soil samples of six glacial meltwater areas in Ny-Ålesund, Arctic, the meltwater area near the reservoir bay had the highest bacterial abundance, and the meltwater area near the sand had the lowest one. The dominant phyla in soil samples were Proteobacteria, Actinobacteria, Acidobacteria. The NH_4_^+^-N content in intertidal soil was higher than that in subtidal soil. Through WGCNA analysis and LEFSE analysis, it was found that the core bacteria significantly related to NH_4_^+^-N were basically distributed in the intertidal area. For example, *Nitrosomonadaceae*, *Nitrospira* and *Sphingomonas* were the core bacteria showed significant different abundance in the intertidal area, which have the ability to metabolize NH_4_^+^-N. Our findings suggest that NH_4_^+^-N plays an important role in soil bacterial community structure in the Arctic meltwater areas.

## 1. Introduction

Climate change has a profound impact on the global landscape, especially in the Arctic, where the direct human impact is very small [1,2]. One of the most significant landscape changes caused by global warming in polar regions is melting ice into lakes or ponds [3], which not only exposes the soil once covered by glaciers, but also submerges some land and creates a wide range of local heterogeneous ecosystems [1,4]. Lakes in permafrost will expand and shrink with the seasons, and even disappear from the Arctic highlands [5]. Lakes, especially newly formed ponds, represent another way in which soil geochemistry may be changed due to global climate change [6].

The increase of lakes and bare soils caused by climate change in the Arctic region will lead to corresponding changes in the ecological environment in this region. As an important part of the ecosystem, microorganisms not only play the role of decomposers, but are also affected by the physicochemical properties of the environment and nutrients. Important physicochemical factors affecting the structure of microbiomes include organic carbon, water content, pH, phosphorus [7,8,9], redox potential and oxidative stress [10,11], nanoparticles [12] and temperature [13]. The change of environment will cause the change of soil microbial species and diversity.

Few previous studies have compared the intertidal soil with the subtidal soil, while most studies in the Ny-Ålesund area have focused on soil, sediment or topsoil in a single characteristic area [14,15,16]. All these studies can only prove the influence of environmental factors on bacterial community at the sampling site, which is not enough to explain the relationship between environmental factors and bacterial community change in Ny-Ålesund area. In this study, six meltwater areas with different characteristics were used to prove the main physicochemical factors of bacterial community changes in the intertidal and subtidal zones of meltwater areas in the Ny-Ålesund area to a certain extent.

In this study, in order to study the differences of soil bacterial communities in intertidal and subtidal zones of different meltwater regions and the influence of soil physicochemical factors, six meltwater areas with different characteristics were selected from the Ny-Ålesund, Arctic and the soil samples of intertidal and subtidal zones were collected. The bacterial diversity composition of soil samples was analyzed by high-throughput sequencing of 16S rRNA V3–V4 gene. Meanwhile, the physicochemical properties of these soil samples were determined by conventional instrumental analysis to understand their influence on the composition of soil bacterial communities and to explore the obvious influence factors.

## 2. Materials and Methods

### 2.1. Sampling Site Description and Sample Collection

The study area is located in the Ny-Ålesund region in the northwest of Svalbard Archipelago in the Arctic. Under the influence of the North Atlantic warm current, the climate in this region is relatively warm and humid. It is one of the regions with the highest temperature in the same latitude in the Arctic, and also one of the regions with the fastest warming in the Arctic [17]. Due to the active glacier erosion, the landforms in the Ny-Ålesund area are mainly divided into four categories, including glacier, moraine landform, flowing water landform, and other types of landforms, while the other types are complex landforms composed of sediments, naked rocks, stone rings, foreign weathering materials, colluvial deposits and coastal landforms [18]. Samples were collected in July 2018 from 36 soil samples from six meltwater areas and12 sites in the Ny-Ålesund region of the Arctic (Table 1). Using a sterile spoon, 50 g of soil, about 5 cm deep, was dug into a sterile bag. Samples collected were temporarily stored at 20 °C and then immediately transferred to a −80 °C ultra-low temperature refrigerator for long-term storage.

### 2.2. Soil Physicochemical Property Analyses

The determination of soil physicochemical properties included 9 items. Soil moisture content (MC) was determined by drying at 105 °C to constant weight. Soil pH was measured by adding 10 mL of distilled water to 4 g of soil and then using a pH meter (PH-3C, Shanghai Rex Instrument Factory, Shanghai, China). OrC and OrN were determined by the following procedure [19]: First, soil samples were lyophilized and ground to powder, then subjected to overnight shock treatment with 10% HCl, washed with ultrapure water and dried at 55 °C, and finally analyzed using an elemental analyzer (EA3000, Euro Vector SpA, Milan, Italy). The determination process of NH_4_^+^-N, SiO_4_^2−^-Si, NO_3_^−^-N, NO_2_^−^-N, and PO_4_^3−^-P was as follows [20]: After lyophilization and grinding to powder, soil samples were added with deionized water at a ratio of 1:10 (g/mL), shaken every 4 h for a total of 48 h, and determined using a nutrition autoanalyzer (QuAAtro, SEAL, Darmstadt, Germany). The relative standard deviation is less than 5%.

### 2.3. DNA Extraction, PCR Amplification, and Sequencing

The total DNA (10–30 ng/μL^−1^) of the soil sample was extracted according to the instructions of the Mobio PowerSoil DNA Isolation Kit (MOBIO, laboratories, Inc., Carlsbad, CA, USA, and QIAGEN company, Germantown, MD, USA) [21]. A 30 µL PCR reaction system of 16S rRNA V3-V4 gene included 15 μL Phusion^®^ high-fi PCR Master Mix (New England Biolabs, Ipswich, MA, USA), 0.2 μmol/L^−1^ of primers 341F and 806R [22,23], 10 ng of template DNA. The PCR reaction conditions were as follows: initial denaturation at 98 °C for 1 min, 30 cycles of denaturation at 98 °C for 10 s, annealing at 50 °C for 30 s, extension at 72 °C for 30 s, and the final extension at 72 °C for 5 min. The PCR products were between 400 bp and 450 bp.

### 2.4. High-Throughput Sequencing and Analysis

High-throughput sequencing of the 16S rRNA gene V3-V4 region was performed on an Illumina MiSeq platform. Clean Data were obtained by concatenation and quality control to remove low-quality sequences. QIIME (Version 1.7.0, Knight and Caporaso labs) [24], UCHIME algorithm [25], and Gold database [26] were used for chimera filtering. The effective sequences were clustered using Uparse software (Version 7.0) [27], then into Operational Taxonomic Units (OTUs) according to 97% similarity. The sequence with the highest frequency in each OTU was selected as the representative sequence, SSUrRNA database [28] was selected as the species annotation library, and the representative sequence of OTU was annotated with the species annotation library to obtain its taxonomic information. The same sequence depth of all samples was then normalized for subsequent alpha diversity analyses.

Software Qiime 1.7.0 [24] was used to statistically analyze the alpha diversities and diversity index of OTUs. R software (Version 3.2.4) [29] was used to conduct a Kruskal-Wallis test on the physicochemical properties of samples and species diversity parameters, and to distinguish bacterial flora at different sampling points [30]. Redundancy analysis (RDA) and the Monte Carlo test were used to explain the correlation between sample physicochemical properties and station [31] distribution. Through the module and physicochemical factor heat map, Weighted Correlation Network Analysis (WGCNA) software (https://www.r-project.org/, accessed on 24 August 2022) package was used to draw the core flora network map and combined with the results of LEfse analysis, the influence of physicochemical factors on the flora structure was analyzed.

## 3. Results

### 3.1. Physicochemical Properties of Soils

**Figure 1:**
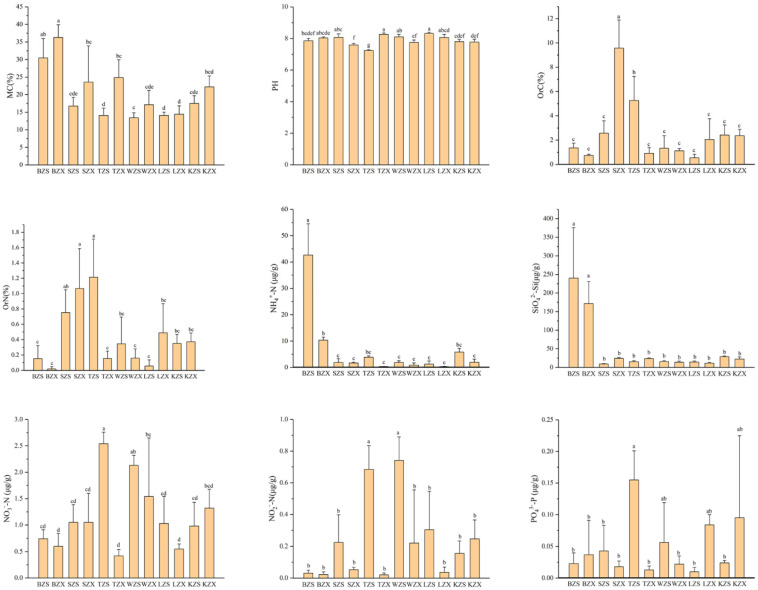
Physicochemical properties of soil samples from the meltwater areas of Ny-Ålesund.

Nine physicochemical properties, including moisture content, pH, organic carbon, organic nitrogen, and five soluble nutrients (ammonium salt, silicon salt, nitrate salt, nitrite salt, and phosphorus salt) of soil samples in 12 sites (BZS, BZX, SZS, SZX, TZS, TZX, WZS, WZX, LZS, LZX, KZS, KZX) were measured (Figure 1). It could be seen from Table 2 that the contents of silicon salt and ammonium salt had higher values in BZS and BZX, the content of nitrate was higher in TZS and WZS, the contents of organic carbon and organic nitrogen were higher in SZX and TZS, and the content of phosphorus salt reached highest in TZS. The differences in these physicochemical factors may lead to different diversity of the bacterial community at these sampling sites.

### 3.2. Bacterial Diversity and Community Composition

**Figure 2:**
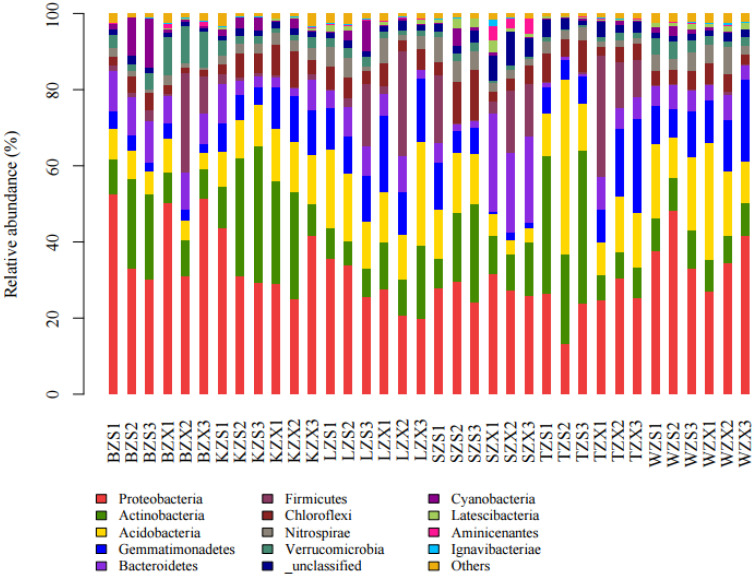
Phyla of the top 15 abundance in 36 soil samples.

**Figure 3:**
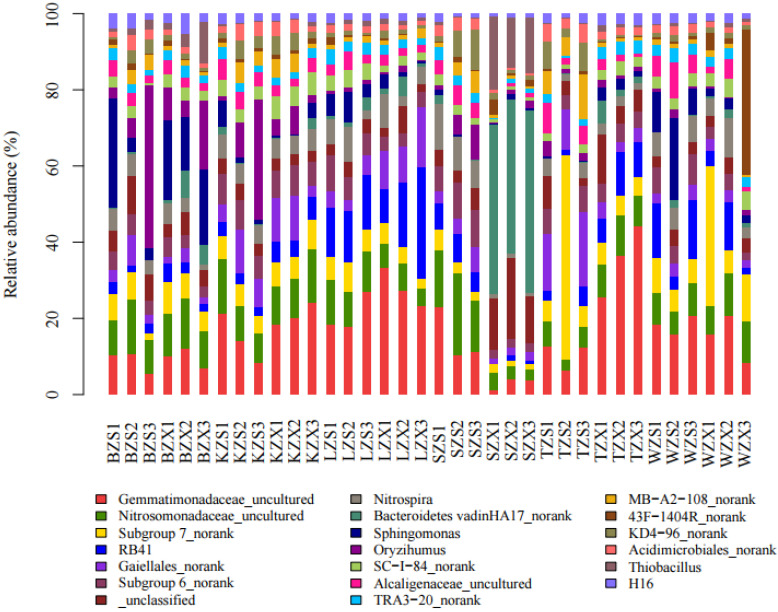
Genus of the top 20 abundance in 36 soil samples.

Thirty-six samples from six meltwater areas in Ny-Ålesund, Arctic were high-throughput sequenced, and an average of 58,012 sequences were obtained from each sample for subsequent analysis. The effective sequences of all samples were clustered into OTU according to the sequence similarity of 97%, and a total of 19,258 OTUs were obtained. On average, there were 3105 OTUs in each sample and 9316 OTUs in each sampling site. OTUs of all samples were clustered into 59 phyla, among which Proteobacteria, Actinobacteria, Acidobacteria, Gemmatimonadetes, Bacteroidetes, and Chloroflexi were the phyla with high relative abundance (Figure 2). Among them, Proteobacteria had the highest abundance in BZS and BZX; The abundance of Actinobacteria in TZS was the highest, and its abundance in the intertidal zone was higher than that of the subtidal zone; Bacteroidetes had the highest abundance in SZX; Firmicutes had the highest abundance in TZX, but the lowest abundance in TZS. Among the top 20 genera in abundance (Figure 3), it was found that each site had unique genera with high relative abundance. For example, the genus with the highest relative abundance at SZX was Bacteroidetes vadinHA17_norank, while its relative abundance at other sites was low. The relative abundance of 43F-1404R at WZX was much higher than that at other sites. In addition, the relative abundance of *Oryzihumus* and Alcaligenaceae uncultured in the intertidal zone was higher than that in the subtidal zone in all sampling sites, while that of *Thiobacillus* was on the contrary.

At the same time, through Alpha diversity analysis, it can be seen that the Good’s coverage index of 36 soil samples ranges from 96.0% to 98.7%, with the sufficient sequencing depth. ACE was in the range of 2169.978–5346.601, Chao1 index was in the range of 2057.329–5212.196, Shannon index was in the range of 4.523–6.985, and Simpson index was in the range of 0.003–0.143. In all samples, the values of Chao1 (2169.978) and ACE (2057.329) of SZX1 were the lowest, and the Shannon index was 5.269, which proved that the community structure of SZX1 was relatively simple. The Shannon index of KZX3 is 6.919, which was larger than that of all samples, and the Simpson index (0.003) of which was the lowest. The values of Chao1 (5212.196) and ACE (5346.601) of KZX3 were the largest, which proved that the microbial diversity of KZX3 was high, and the population was rich and evenly distributed.

### 3.3. Correlation between Soil Physicochemical Factors and Bacterial Community Structure

**Figure 4:**
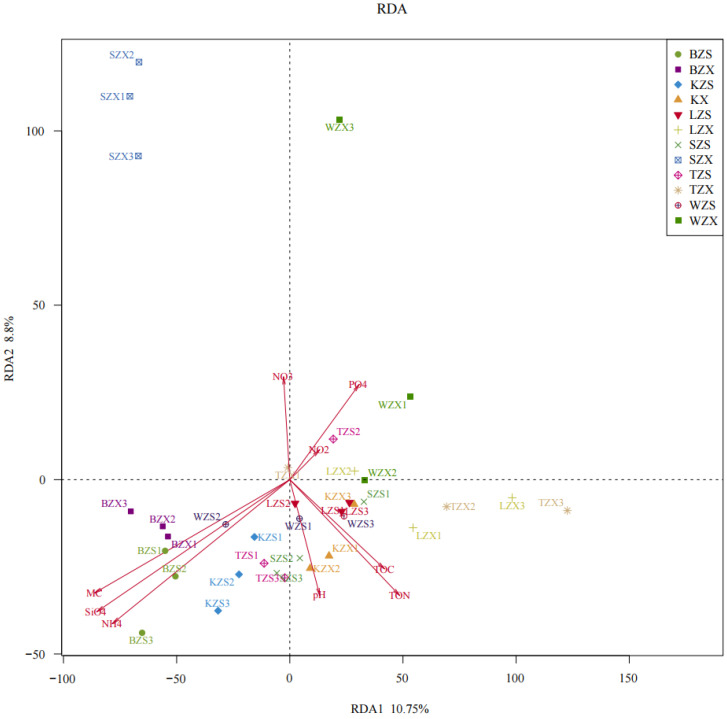
RDA showed that the correlation between bacterial communities and soil physicochemical factors of soil samples.

In this study, redundancy analysis (RDA) was used to explore the relationship between bacterial diversities and nine soil physicochemical factors. The results showed that NH_4_^+^-N, SiO_3_^2−^-Si and MC had great influence on the overall diversity and distribution of bacterial communities. Among all sampling sites, BZS and BZX were most affected by three physicochemical factors (Figure 4). At the same time, it was further verified by Monte Carlo test that the MC (r^2^ = 0.3396, Pr = 0.003), SiO_3_^2−^-Si (r^2^ = 0.3571, Pr = 0.004) and NH_4_^+^-N (r^2^ = 0.321, Pr = 0.005) were significantly correlated with the bacterial communities of soil samples (Table 2).

**Figure 5:**
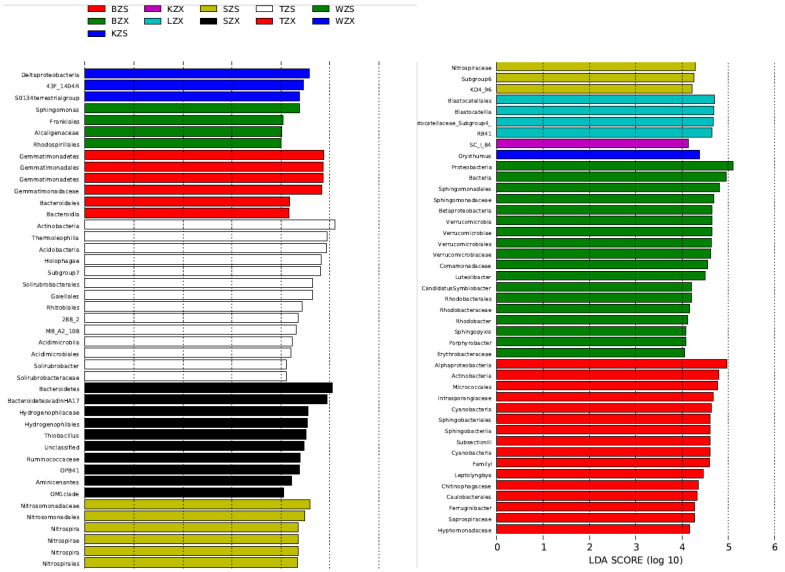
The LDA score showed that the bacterial communities at different sampling sites had different relative abundance.

**Figure 6:**
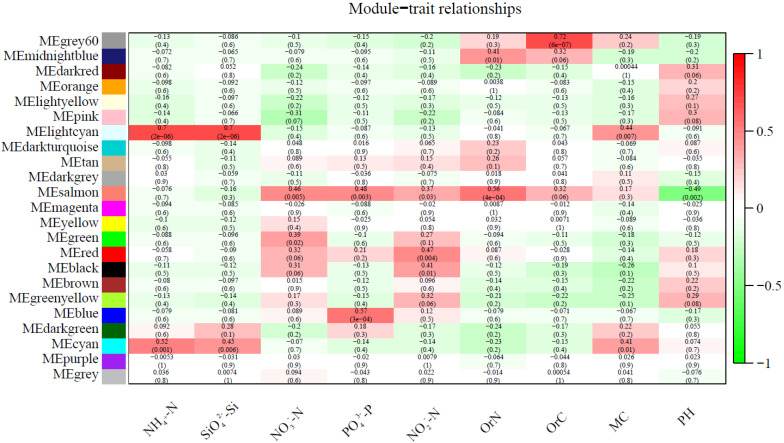
Modules of OTUs associated with nine examined physicochemical factors.

**Figure 7:**
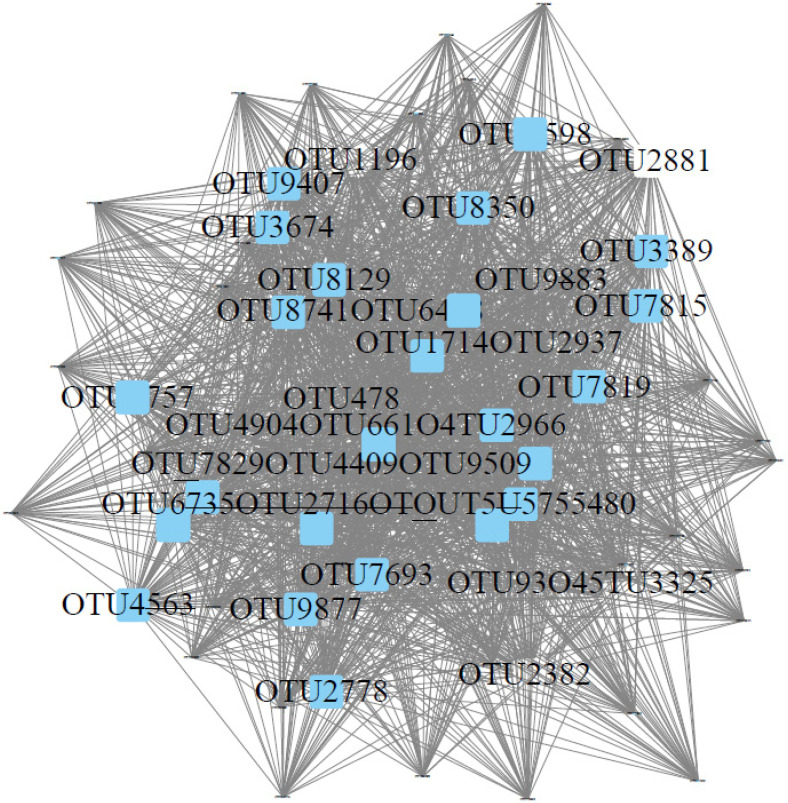
The topology of the MElightcyan module associated with NH_4_^+^-N.

In addition to physicochemical factors, we also focused on bacteria with large differences in bacterial abundance at each sampling site. According to the results of LEfSe, there were significant differences in bacteria at 11 of the 12 sampling sites (Figure 5), among which there were more groups in SZX, SZS, BZX, BZS and TZS, followed by TZX, LZX, WZS and WZS, and KZX and KZS were the least. Weighted Correlation Network Analysis (WGCNA) was performed on all OTUs and physicochemical factors of sampling sites, and the related heat maps (Figure 6) of the modules and physicochemical factors were obtained. Module MElightcyan had a strong positive correlation with NH_4_^+^-N and SiO_3_^2−^-Si. The results obtained from module MElightcyan was imported into the Cytoscape to get a network diagram (Figure 7). There were 30 core genera in this picture, including *Nitrosomonadaceae*, *Nitrospira*, *Sphingomonas*, *Hyphomicrobium*, *Leptolyngbya*, *Saprospiraceae*, *Phormidium*, *Sphingobacteriaceae*. Eight of them belong to Proteobacteria and seven to Acidobacteria, which accounted for half of the core genera. In the core bacteria genera, the bacteria with large differences in abundance all belong to the intertidal zone. 

## 4. Discussion

Our study shows that the structure and diversity of bacterial community of the soils in the Ny-Ålesund meltwater area varied with sampling sites and physicochemical factors. In this study, the high level of Good’s Coverage index (96.0–98.7%) and the identification of 19,258 OTUs reveal a surprising diversity of bacterial communities in these meltwater areas. From high-throughput sequencing data of the 16S rRNA V3-V4 gene region, we found that most bacterial taxonomic groups belong to common phyla, including Proteobacteria, Actinobacteria, Acidobacteria, Gemmatimonadetes, Bacteroidetes, Chloroflexi. Proteobacteria, Actinobacteria and Acidobacteria, with relatively high abundance, were also frequently found in low temperature environments besides the Arctic, such as the Antarctic, the Tibetan Plateau and Siberia [31,32,33,34]. It indicates that these bacteria have good adaptability to low temperature environment. The relative abundance of these three common bacterial phyla was the highest, which was consistent with previous literature [35,36,37]. Although the first 15 phyla were shared between all sampling sites, their abundance varied. For example, compared with other sites, the abundance of Bacteroidetes in SZS is the highest, but that abundance of Gemmatimonadetes in SZS was very low. 

In the Arctic, physicochemical factors play an important role in the change of soil bacterial community diversity. Although we observed some differences in physicochemical factors in soil samples from meltwater areas with different characteristics, MC (R2 = 0.3396, Pr = 0.003), SiO_3_^2−^-Si (R2 = 0.3571, Pr = 0.004) and NH_4_^+^-N (R2 = 0.321, Pr = 0.005) were significantly correlated with the bacterial communities in all the six meltwater areas. It is obvious that among them, the moisture content in the subtidal area was higher than that in the intertidal area. The high correlation of SiO_3_^2−^-Si may be caused by erosion and leaching of sedimentary rocks and sand by meltwater at the glacial front, forming a high concentration at the bottom of the meltwater area [34]. However, the content of ammonium in intertidal area was higher than that in subtidal area in all meltwater areas. The change of bacterial community caused by the change of physicochemical factors can be used as an indicator of the short-term and long-term effects of land submergence caused by global warming. Some compositional changes have been noted in existing work on thawed lakes in the Arctic region [14] and in our previous studies [38]. The general continuity between the intertidal and subtidal zones in the meltwater zone suggests that changes in meltwater coverage affect microbial communities in both subtidal and intertidal zones. 

In addition, local soil factors, rather than global factors, have been identified as the main direct formative force of the Arctic microbiome [39]; local soil factors include pH, moisture content, carbon, nitrogen, phosphorus and trace elements [7,8,9,40]. Ammonium and SiO_4_^2−^-Si were two main influential factors determined by network analysis. Most importantly, we revealed the physicochemical factors influencing the intertidal and subtidal microbial communities by establishing relationships between subnetworks and physicochemical factors, and by identifying core bacterial communities significantly associated with ammonium and their main sampling sites. Among all the dominant bacteria, Proteobacteria was the most abundant one, among which ammonia-oxidizing bacteria consume NH_4_^+^-N at a slower rate, and the adsorption capacity of NH_4_^+^-N is stronger in the intertidal area. Therefore, it was observed that the NH_4_^+^-N content in the intertidal areas was higher than that in the subtidal areas [41]. Although we have not determined the core bacteria genera affected by SiO_3_^2−^-Si, we found that the core bacteria genera which were strongly correlated with NH_4_^+^-N had significant differences in the intertidal area combined with the bacteria with significant different abundance at sampling sites analyzed by LEFSE. For example, the core bacterial genera with significant differences in SZS belong to *Nitrosomonadaceae* and *Nitrospira*. *Nitrosomonadaceae* belongs to β-proteobacteria and is a kind of aerobic ammonia-oxidizing bacteria [42] that perform chemoautotroph and mainly act on ammonium nitrogen to oxidize them to nitrite, and it is usually used in sewage treatment systems. *Nitrospira* is an ammonia-oxidizing bacteria that uses AMO and HAO [43,44] to oxidize ammonia to nitrate [42]. *Sphingomonas*, the core bacteria with significant difference in WZS, is abundant in marine sediments and terrestrial soil, and often plays an important role in the denitrification process of industrial wastewater (containing nitrogen) and domestic sewage and the pollution removal of contaminated water sources [45]. The module-related physicochemical factors in the network analysis performed in this study are consistent with the important factors obtained in the RDA analysis, and the two methods complement each other by identifying the primary influence of NH_4_^+^-N on changes of intertidal and subtidal bacterial communities in both local (module) and global (all OTUs) aspects.

## 5. Conclusions

We observed significant differences in physicochemical factors and bacterial community diversity between intertidal and subtidal areas and between different meltwater areas. NH_4_^+^-N is an important explanatory factor for intertidal and subtidal microbial communities. Although different meltwater areas have different physicochemical factors and dominant bacterial communities due to their different ecological environment, NH_4_^+^-N is the influential factor of the bacterial community in intertidal and subtidal areas in all meltwater areas. As global warming intensifies, lakes and ponds of arctic glacial meltwater will gradually increase, so changes in physicochemical factors and microbial structures will become increasingly important.

## Figures and Tables

**Table 1:** Sample collection sites.

Sample Code	Location Profile	Coordination	
BZS	Intertidal soil in the meltwater area near the Glacier	78.9036° N	12.0447° E
BZX	Subtidal soil in the meltwater area near the Glacier		
SZS	Intertidal soil in the meltwater area near the Sand	78.95779° N	11.7050° E
SZX	Subtidal soil in the meltwater area near the Sand		
TZS	Intertidal soil in the meltwater area near the Tundra	78.9652° N	11.6013° E
TZX	Subtidal soil in the meltwater area near the Tundra		
WZS	Intertidal soil in the meltwater area near the Bay	78.9664° N	11.5792° E
WZX	Subtidal soil in the meltwater area near the Bay		
LZS	Intertidal soil in the meltwater area near the Lake London	78.9626° N	12.0592° E
LZX	Subtidal soil in the meltwater area near the Lake London		
KZS	Intertidal soil in the meltwater area near the Reservoir bay	78.9194° N	11.8766° E
KZX	Subtidal soil in the meltwater area near the Reservoir bay		

**Table 2:** Monte Carlo test of the relationship between soil physicochemical factors and bacterial compositions.

	RDA1	RDA2	r^2^	Pr (> r)	
MC	−0.825606	−0.564247	0.3396	0.003	**
PH	0.139400	−0.990236	0.0512	0.427	-
OrC	0.806820	−0.590797	0.0739	0.261	-
OrN	0.756620	−0.653855	0.1074	0.138	-
PO_4_^3−^-	0.634393	0.773011	0.0784	0.234	-
NH_4_^+^-N	−0.761526	−0.648134	0.3321	0.005	**
SiO_3_^2−^-Si	−0.796176	−0.605065	0.3571	0.004	**
NO_3_^−^-N	0.087895	0.996130	0.0395	0.549	-
NO_2_^−^-N	0.715481	0.698633	0.0109	0.864	-

Note: * Correlation is significant at the 0.05 level. ** Correlation is significant at the 0.01 level.

## Data Availability

Not applicable.
